# Genome-wide CRISPR/Cas9 screening identifies CARHSP1 responsible for radiation resistance in glioblastoma

**DOI:** 10.1038/s41419-021-04000-3

**Published:** 2021-07-21

**Authors:** Guo-dong Zhu, Jing Yu, Zheng-yu Sun, Yan Chen, Hong-mei Zheng, Mei-lan Lin, Shi Ou-yang, Guo-long Liu, Jie-wen Zhang, Feng-min Shao

**Affiliations:** 1 Departments of Geriatrics and Oncology, Guangzhou Geriatric Hospital, 510180 Guangzhou, China; 2 Departments of Geriatrics and Oncology, Guangzhou First People’s Hospital, School of Medicine, South China University of Technology, 510180 Guangzhou, China; 3 Department of Blood Transfusion, Wuhan No.1 Hospital/Wuhan Hospital of Traditional Chinese and Western Medicine, Tongji Medical College, Huazhong University of Science and Technology, 430022 Wuhan, China; 4 Department of Neurology, Henan Provincial People’s Hospital, Zhengzhou University People’s Hospital, 450003 Zhengzhou, Henan China; 5 Department of Geriatrics, The First Affiliated Hospital of Chongqing Medical University, 400016 Chongqing, China; 6 Department of Breast Surgery, Hubei Cancer Hospital, Tongji Medical College, Huazhong University of Science and Technology and Hubei Provincial Clinical Research Center for Breast Cancer, 430079 Wuhan, China; 7 Department of Infection Disease, The Fifth Affiliated Hospital of Guangzhou Medical University, 510150 Guangzhou, China; 8 Department of Nephrology, Henan Provincial Key Laboratory of Kidney Disease and Immunology, Henan Provincial People’s Hospital, Zhengzhou University People’s Hospital, 450003 Zhengzhou, Henan China

**Keywords:** Tumour biomarkers, Glial biology

## Abstract

Glioblastomas (GBM) is the most common primary malignant brain tumor, and radiotherapy plays a critical role in its therapeutic management. Unfortunately, the development of radioresistance is universal. Here, we identified calcium-regulated heat-stable protein 1 (CARHSP1) as a critical driver for radioresistance utilizing genome-wide CRISPR activation screening. This is a protein with a cold-shock domain (CSD)-containing that is highly similar to cold-shock proteins. CARHSP1 mRNA level was upregulated in irradiation-resistant GBM cells and knockdown of CARHSP1 sensitized GBM cells to radiotherapy. The high expression of CARHSP1 upon radiation might mediate radioresistance by activating the inflammatory signaling pathway. More importantly, patients with high levels of CARHSP1 had poorer survival when treated with radiotherapy. Collectively, our findings suggested that targeting the CARHSP1/TNF-α inflammatory signaling activation induced by radiotherapy might directly affect radioresistance and present an attractive therapeutic target for GBM, particularly for patients with high levels of CARHSP1.

## Introduction

Glioblastomas (GBM) is the most common primary malignant brain tumor in adults, with a high propensity of invasion and proliferation [1, 2]. GBM patients are subjected to an aggressive therapy regimen comprising maximal save resection, radiotherapy (50–60 Gy), and administration of temozolomide [3, 4]. Radiotherapy plays a critical role in the curative management of patients with inoperable malignancies [5]. Unfortunately, populations of glioma stem-like cells and infiltrating tumor cells can survive radio-chemotherapy, acquire additional mutations, and be resistant to therapy [6–8], which leads to the 5-year survival rate of GBM remains under 10% [5]. Oncologists have sought to identify novel molecular targets involved in resistance to improve therapy for GBM. Zhang et al’s study [9] showed that depletion of TAZ by RNAi promoted radiation-induced senescence and growth arrest, which might benefit GBM radiotherapy. Some studies [2] supported that induction of ER stress signaling by radiation contributes to adaptive survival mechanisms during radiation therapy.

Radioresistance occurs through various cellular conditions including survival signaling activation, hypoxic microenvironments, and inflammatory responses [10–13]. And inflammation played a pivotal role in modulating the radiation responsiveness of cells [14]. Calcium-regulated heat-stable protein 1 (CARHSP1), also known as CRHSP-24, has been identified as a cold-shock domain (CSD) protein family member, functions as a transcriptional or translational regulator [15, 16]. CARHSP1 contains a CSD, making it possible for it to bind to polypyrimidine regions and regulate the stability of single-stranded RNA or DNA [17, 18]. Recently, it was found to be an mRNA stability enhancer for tumor necrosis factor-alpha (TNF-α), which is the central mediator of inflammation in macrophages, and several studies have demonstrated that CARHSP1 binds to the AU-rich element (ARE) of the TNF-α 3′-UTR through the CSD [19–21].

A number of traditional or novel drugs and natural products have shown their potent properties for radiosensitizers to block the inflammatory signaling pathway in cases of cancer [22, 23]. Seeking vulnerabilities of radioresistance targets in GBM is a promising strategy to improve the efficiency of radiotherapy. Through genome-scale gain-of-function screening, we found a new radioresistance target CARHSP1, which was amazingly overexpressed in radiotherapy-resistant GBM cells. In summary, we determined that the increased CARHSP1 level might play a radiotherapy protective role by signaling through the CARHSP1/TNF-α pathway.

## Materials and methods

### Cell culture

The human glioblastoma U87 and U251 cell lines were obtained from Procell Life Science & Technology Co., Ltd (Wu Han, China) and were cultured in Dulbecco’s modified Eagle’s medium (DMEM, HyClone, SH30022.01B), with 10% fetal bovine serum (FBS, Gibco) and 0.1% Penicillin/Streptomycin (P/S, TBD, PS2004HY) in a humidified incubator with 5% CO_2_ at 37 °C.

### Measurement of minimum radiation dose

Radiation of cells was performed at a dose rate of 0, 3, 6, 9, 12, 15, and 18 Gy/min with 6 mV Xray Irradiator using a Linear Accelerator, Elekta synergy. The growth of GBM cells was observed by an optical microscope (Nikon) and the OD value were then detected by CCK8 assay to determine the minimum lethal dose (MLD) of radiation.

### Pooled genome-wide CRISPR screening

In this study, the Human CRISPR activation pooled library (Purchased from Addgene, #100000007, #89308) was used to identify genes responsible for radiation resistance in GBM cells. According to the CCK8 assay, the inhibition of cell growth was much more significant at a dose rate of 12 Gy/min (U87 cells) and 15 Gy/min (U251 cells). Therefore, 12 Gy/min (U87 cells) and 15 Gy/min (U251 cells) radiation doses were chosen in the following experiments. We first transfected the U87 and U251 cells with the activator plasmid MS2-P65-HSF1, selected with hygromycin B (YEASEN, 60225ES03). Then at least 1 × 10^8^ U87 and U251 cells were transduced with pooled CRISPR/Cas9 SAM human lentiviral library which contains 70,290 unique sgRNA sequences targeting 23,430 human genes (3 sgRNAs per gene) at a low MOI (~0.5) to ensure most cells received only one stably integrated RNA guide. The transduced cells were selected with blasticidin (MDBio, D0120601) for 15 days to generate a mutant cell pool, which was then treated with 12 Gy/min (U87 cells) or 15 Gy/min (U251 cells) radiation for three rounds, respectively. After treatment, these resistant cells were collected for genomic DNA extraction and deep sequencing analysis.

### Genomic DNA extraction and sgRNA deep sequencing

The HiPure Tissue DNA Mini Kits (Magen) were used to extract genomic DNA. Amplification of the sgRNA sequences of each sample from the extracted genomic DNA using the CRISPRa-F: TCTTGTGGAAAGGACGAAACACCG and CRISPRa-R:CTCCTTTCAAGACCTAGGATC primers. The thermocycling parameters were: 95 °C for 60 s, followed by 35 cycles of (95 °C for 10 s, 60 °C for 10 s, and 72 °C for 30 s) and a final extension at 72 °C for 1 min. After the PCR products were electrophoresed, HiPure Gel Pure DNA Mini Kits were used for gel extraction. The pooled products were gel purified from a 2% E-gel EX (Life, Technologies) using the QiaQuick kit (Qiagen). The purified pooled library was then selected by agarose gel and subjected to massive parallel amplicon sequencing carried out by Novogene Technology (Beijing, China).

### Screen enrichment analysis

The R software package (DESeq2) was applied to perform a statistical analysis of sequencing data. The Kyoto Encyclopedia of Genes and Genomes (KEGG) Orthology-Based Annotation System (KOBAS) 3.0 (http://kobas.cbi.pku.edu.cn/anno_iden.php) was used to identify significant biological pathways in the CRISPR library datasets.

### TCGA data analysis

For 529 GBM samples in TCGA, mRNA seq and clinical data were retrieved from UCSC Xena (https://xenabrowser.net/datapages/) and convert FPKM data to TPM. Finally, 498 patients with mRNA expression data and prognosis information were divided into high expression group and low expression group according to the median (4.6978), and then Kaplan–Meier survival analysis and univariate Cox hazard analysis were performed. Finally, 139 patients in TCGA treated with radiotherapy were divided into high expression group and low expression group according to the same median, and then Kaplan–Meier survival analyses were performed.

### Lentiviral transduction

The pLenti-pLKOG-KIAA0895, pLenti-pLKOG-STRA6, pLenti-pLKOG-FBLIM1, pLenti-pLKOG-CARHSP1 and pLenti-pIRES2-EGFP-KIAA0895, pLenti-pIRES2-EGFP-STRA6, pLenti-pIRES2-EGFP-FBLIM1, pLenti-pIRES2-EGFP-CARHSP1 plasmids were obtained from GENERAL BIOL (Chuzhou, China). Lentiviruses were generated by co-transfecting the target plasmid and the packaging plasmid (pCMV-VSV-G, pMDLg pRRE, and 3 μg pRSV-Rev) into 293T cells using polyethyleneimine (PEI). U251 and U87 cells were seeded in 6-well plates and incubated with virus supernatant and observe the fluorescence at 48 h, after which they were screened with 5 μg/ml puromycin for 48 h. Puromycin-resistant cells were then used in downstream assays.

### Cell proliferation assay

We detected the effect of different radiation doses on GBM cells transfected with STRA6, FBLIM1, KIAA0895, CARHSP1 overexpression, and knockdown plasmids by CCK8 assay (Beyotime, Shanghai, China) according to the manufacturer’s instructions.

### Quantitative reverse transcriptase PCR

The mRNA levels of STRA6, FBLIM1, KIAA0895, and CARHSP1 (Supplementary Table S1 for primer sequences) were detected by real-time quantitative reverse transcriptase PCR (qRT-PCR). Total RNA was extracted using the total RNA Rapid Extraction Kit (BIOTEKE, RP1201) according to the manufacturer’s instructions. The SYBR Green ® Realtime PCR Master Mix (#QPK-201, Toyobo Co, Ltd, Osaka, Japan) was used for qRT-PCR assays. The data were analyzed on an Applied Biosystems 7900 Real-Time PCR System. The target genes were normalized to the mean β-actin expression.

### Immunohistochemistry

Expression pattern and subcellular localization of CARHSP1 (Abcam, ab96677, 1:300) protein in clinical GBM tissues were detected by immunohistochemistry. Tumor specimens (Xi ‘an Alina Biotechnology Co., Ltd, GL803c) were scored in a semiquantitative manner due to the heterogeneity of the staining of the target proteins as described previously [24]. A final immunoreactivity scores (IRS) was obtained for each case by products of the percentages and the intensity scores.

### Statistical analysis

Our data were expressed as means ± SD. Analyses were performed using IBM SPSS Statistics for Windows, version 20.0 (IBM Corp., Armonk, NY, USA). Comparisons between groups were analyzed through a one-way analysis of variance (ANOVA). *P*-values less than 0.05 were considered statistically significant.

## Results

### A whole-genome CRISPR screen identifies mediators of irradiation resistance

**Fig. 1:**
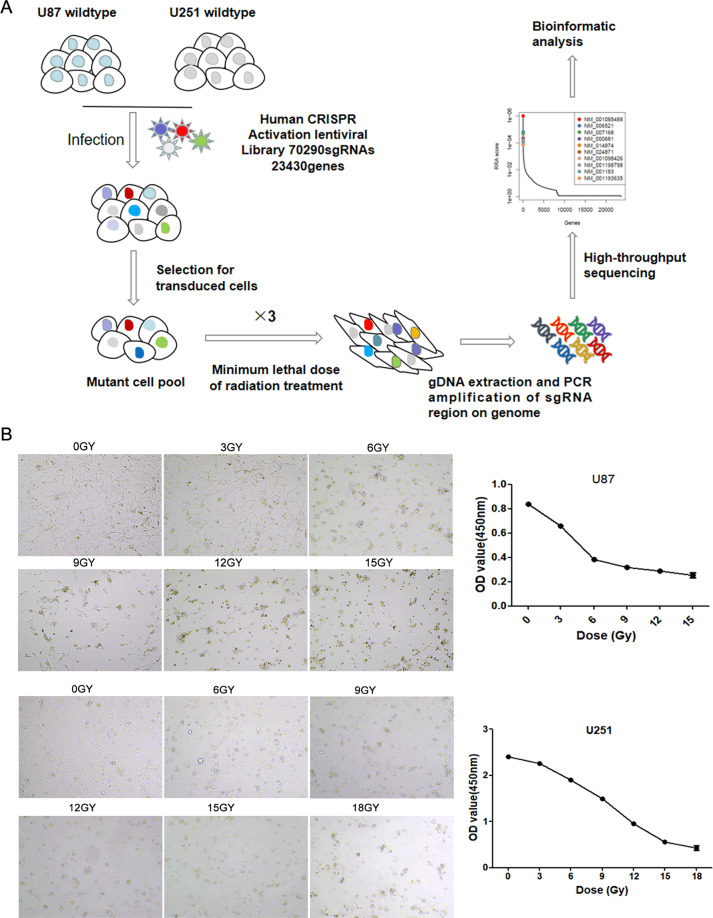
Schematic of functional screening by CRISPR/Cas9 SAM pooled library and irradiation treatment. **A** Schematic of irradiation-resistant GBM cells construction for high-throughput sequencing analysis. **B** U87 (0, 3, 6, 9, 12, 15 Gy/min) and U251 (0, 6, 9, 12, 15, 18 Gy/min) cell lines were treated with the indicated doses of metformin for 24 h, and cell viability was measured using an CCK8 assay. Data were represented as means ± SD of a representative experiment (of three experiments).

In this study, the human CRISPR activation pooled library, which included 70,290 sgRNAs targeting 23,430 protein-coding genes in the human genome was used to identify genes responsible for irradiation resistance in GBM cells. Figure 1A displayed a schematic of irradiation-resistant GBM cells enrichment for high-throughput sequencing analysis. To measure the minimum radiation dose of GBM cells to radiation, we treated human U251 and U87 cell lines with different doses of gamma radiation. The CCK8 assay indicated a dose-dependent growth inhibition by radiation. According to the result (Fig. 1B), few U87 cells survived at day 6 following treatment with 12 Gy/min irradiation, and all cells died with 15 Gy/min radiation, few U251 cells survived at day 5 following treatment with 15 Gy/min radiation and all cells died with 18 Gy/min irradiation. Therefore, 12 Gy/min (U87 cells) and 15 Gy/min (U251 cells) radiation doses were chosen in the following experiments.

### Enriched genes in irradiation-resistant GBM cells

**Fig. 2:**
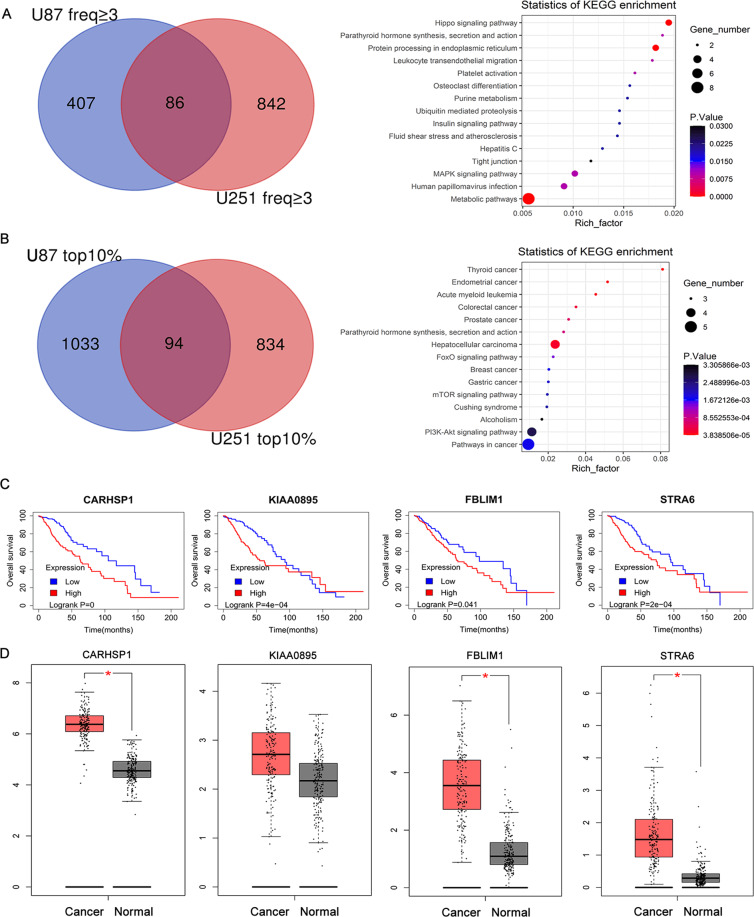
Enriched irradiation-resistant genes from the genome-scale CRISPR/Cas9 activation screen in GBM. **A** Significantly enriched Kyoto Encyclopedia of Genes and Genomes (KEGG) pathways for genes with sgRNA diversity >3 in irradiation-resistant GBM cells. **B** The genes with the top 10% of sgRNA abundance in GBM cells (Supplementary Table S2) were intersected and were then used for KEGG pathway analysis. **C** Kaplan–Meier analysis indicated that GBM patients with high expression of CARHSP1, KIAA0895, FBMIL1, and STRA6 exhibited worse overall survival. **D** CARHSP1, FBMIL1, and STRA6 mRNA expression were selectively upregulated in GBM compared with the Normal (GEPIA).

The mutant cell pool was treated with 12 Gy/min (U87 cells) or 15 Gy/min (U251 cells) radiotherapy for three rounds, respectively, and the surviving cells were enriched (Supplementary Fig. S1). The survival resistant cells were cultured and collected to extract DNA and PCR amplification of the 209-bp sgRNA region was used for high-throughput sequencing to calculate the sgRNAs coverage in the cells. The genes with the top 10% of sgRNA abundance in U87 and U251 cells (Supplementary Table S2) were intersected and the 86 genes were then used for Kyoto encyclopedia of genes and genomes (KEGG) pathway analysis to determine the processes that mediated irradiation resistance. Figure 2A showed significant enrichment of 15 pathways, including metabolic pathways, Hippo signaling pathway, Protein processing in the endoplasmic reticulum, MAPK signaling pathway, and so on. We further analyzed genes with sgRNA diversity >3 (Supplementary Table S2) in U87 and U251 cells and 94 genes were enriched (Fig. 2B). The result showed these genes were enriched in the FoxO signaling pathway, Pathways in cancer, mTOR signaling pathway, PI3K-Akt signaling pathway, etc (Fig. 2B). There are 15 genes both with top 10% of sgRNA abundance and sgRNA diversity greater than 3 (freq > 3) in U251 and U87 cells. Finally, the TCGA data were combined to further analyze the 15 genes, 4 of which affect the prognosis of GBM (*P* < 0.01).

We analyzed a total of 498 patients with mRNA expression data and prognosis information, then the samples were divided into high expression group and low expression group according to the median. Kaplan–Meier survival analysis and univariate Cox hazard analysis were performed. The Kaplan–Meier analysis indicated that GBM patients with high expression of CARHSP1, KIAA0895, FBMIL1, and STRA6 exhibited worse overall survival as compared to patients with low expression of these genes (*P* = 0.0, *P* = 4e−04, *P* = 0.041, *P* = 2e−04, log-rank test, Fig. 2C). Of the identified hits, CARHSP1, FBMIL1, and STRA6 mRNA expression was selectively upregulated in GBM compared with the Normal (*P* < 0.05) (Fig. 2D) (GEPIA, Gene Expression Profiling Interactive Analysis). These results suggested high expression of CARHSP1, KIAA0895, FBMIL1, and STRA6 not only leads to irradiation-resistant but also affects the prognosis of GBM.

### Overexpression of candidate genes promoted GBM cells resistance to radiotherapy

**Fig. 3:**
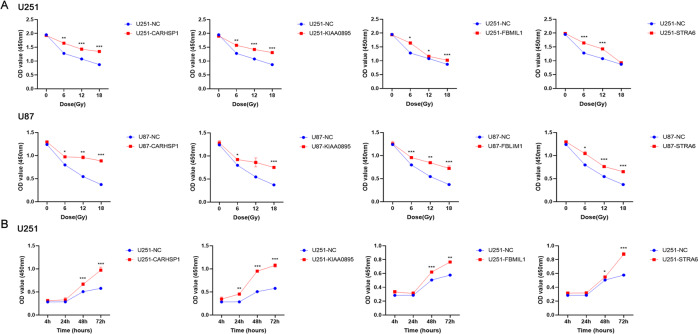
The overexpression of the candidate genes increases irradiation resistance in GBM cell lines. **A** U87 and U251 cell lines with single gene overexpression were treated with 0, 6, 12, and 18 Gy/min irradiation, and cell viability was measured using a CCK8 assay. **B** Time-course cell proliferation experiments (4 h, 24, 48 h, 72 h) under 12 Gy/min irradiation in U251-NC, U251-CARHSP1, U251-KIAA0895, U251-FBMIL1, and U251-STRA6 cells were detected by CCK8. Data were represented as means ± SD of three independent experiments.

To validate the function of the candidate genes identified in the CRISPRa screen, we overexpressed the individual genes in U251 and U87 cells. The qPCR assay confirmed the high expression of these genes (Supplementary Fig. S2). We then tested their sensitivity of these cells to irradiation by CCK8 assays. As shown in Fig. 3A, after treatment with 6, 12, and 18 Gy/min irradiation the decrease of OD value in CARHSP1, KIAA0895, and FBMIL1 overexpression group were significantly impaired compared to those in the non-targeted control (*P* < 0.001). This means that high expression of CARHSP1, KIAA0895, and FBMIL1 gene promoted the proliferation of GBM cells under radiotherapy. Moreover, the time-course cell proliferation experiments under 12 Gy/min irradiation showed overexpression of CARHSP1, KIAA0895, FBMIL1, and STRA6 could significantly promote cell proliferation. Supporting the view that gain-of-function of the candidate genes in GBM cells revealed irradiation resistance (Fig. 3B).

### Knockdown of the candidate genes sensitized GBM cells to irradiation

**Fig. 4:**
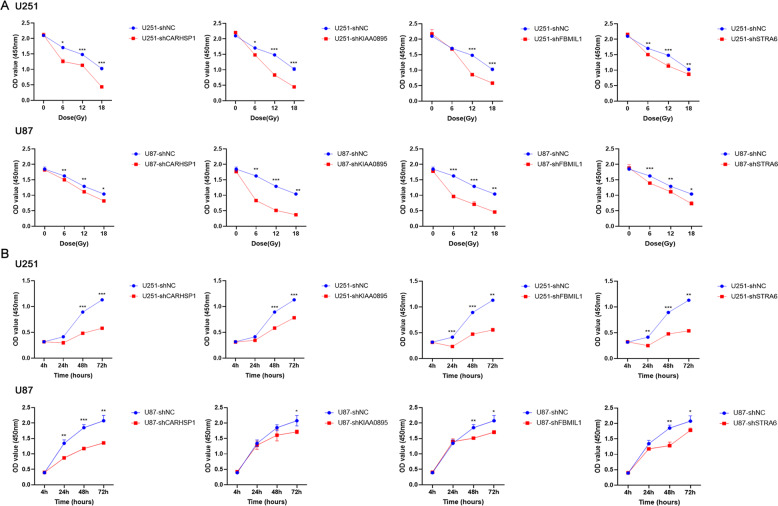
Knockdown of the candidate genes sensitized GBM cells to radiotherapy. **A** U87 and U251 cell lines with single gene knockdown were treated with 0, 6, 12, and 18 Gy/min irradiation, and cell viability was measured using a CCK8 assay. **B** The U87 and U251 cell lines were transfected with CARHSP1, KIAA0895, FBMIL1, and STRA6 knockdown plasmid. Time-course cell proliferation experiments (4 h, 24, 48 h, 72 h) under 12 or 15 Gy/min irradiation were detected by CCK8. Data were represented as means ± SD of three independent experiments.

CARHSP1 [21] contains a cold-shock domain with two RNA binding motifs, which preferentially bind polypyrimidine regions of single-stranded RNA and DNA and regulate ribosomal translation, mRNA degradation, and the rate of transcription termination. We hypothesized high levels of CARHSP1, KIAA0895, FBMIL1, and STRA6 may promote tumor progression. Thus, we investigated whether inhibition of these genes sensitized GBM cells to irradiation treatment. Cell proliferation assay showed that downregulation of CARHSP1, KIAA0895, FBMIL1, and STRA6 could significantly inhibit U87 and U251 cell proliferation when treated with different doses of irradiation (*P* < 0.05, *P* < 0.01) (Fig. 4A). The time-course cell proliferation experiments with 15 or 12 Gy/min irradiation also showed that knockdown of candidate genes could significantly increase the sensitivity of GBM cells to radiotherapy (*P* < 0.05, *P* < 0.01, *P* < 0.001) (Fig. 4B). Taken together, the result demonstrated that inhibition of CARHSP1, KIAA0895, FBMIL1, and STRA6 sensitized GBM cells to irradiation treatment.

### The candidate genes upregulated in irradiation-resistant GBM cells

**Fig. 5:**
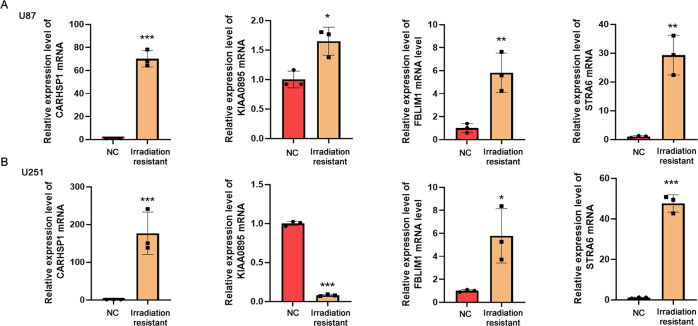
Quantitative real-time PCR analysis for mRNA levels of the candidate genes in wild-type GBM cells and irradiation-resistant GBM cells. **A** CARHSP1 mRNA level in U87 cells was detected, the irradiation-resistant cells of U87 cells were enriched by treating with 12 Gy radiation for three rounds. **B** CARHSP1 mRNA level in U251 cells was detected, the irradiation-resistant cells of U251 cells were enriched by treating with 15 Gy radiation for three rounds. Data were represented as means ± SD of three independent experiments.

Furthermore, the U251 and U87 cells were treated with a minimal lethal dose of radiation. After three rounds of treatment, the irradiation-resistant cells are enriched. Then qPCR was applied to determine the expression levels of CARHSP1, KIAA0895, FBMIL1, and STRA6 mRNAs in U251 and U87 cells and irradiation-resistant GBM cells. As shown in Fig. 5(A-B), the expression levels of CARHSP1, FBMIL1, and STRA6 mRNAs were significantly higher in irradiation-resistant GBM cells than that in wild-type GBM cells (*P* < 0.05). These 3 genes (CARHSP1, FBMIL1, and STRA6) might be the potential genes for irradiation resistance, while KIAA0895 showed an uncertain relationship with irradiation resistance.

### CARHSP1 facilitates irradiation resistance through promoting TNFα in vitro

**Fig. 6:**
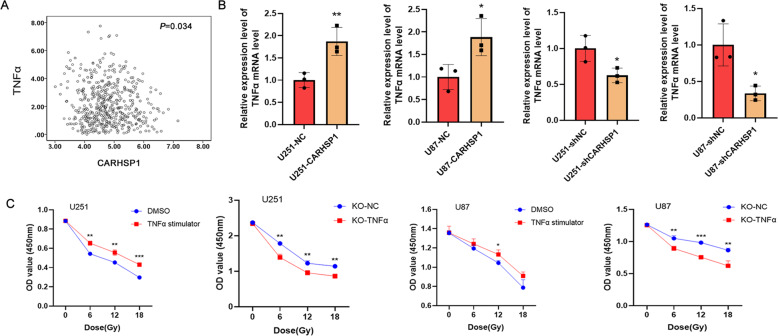
Inhibition of TNF-α could increase the sensitivity of GBM cell to radiotherapy. **A** Correlation analysis of CARHSP1 and TNF-α by the mRNA data in TCGA. **B** qPCR was used to detect the TNF-α mRNA level in CARHSP1 knockdown and overexpression GBM cells. **C** The U87 and U251 cells were treated with TNFα stimulator (Resiquimod, 1 μM) for 24 h or transfected with TNFα knockout plasmid. CCK8 assay was used to measure cell viability of TNFα stimulator and knockout cells when treated with 0, 6, 12, and 18 Gy/min irradiation. Data were represented as means ± SD of three independent experiments.

It was reported that CARHSP1 was specifically interacting with the 3′UTR of TNF-α and knockdown of CARHSP1 inhibited TNF-α protein production in lipopolysaccharide (LPS)-stimulated cells and reduced the level of TNF-α mRNA in both resting and LPS-stimulated cells [21]. Correlation analysis of the TCGA data showed that CARHSP1 mRNA level is positively correlated with TNF-α (*P* = 0.034) (Fig. 6A), which was proved in CARHSP1 knockdown and overexpression GBM cells by qPCR (*P* < 0.05) (Fig. 6B). We deduced high level of CARHSP1 could lead to irradiation resistance by promoting the expression of TNF-α. Therefore, the CCK8 assay was used to prove whether the TNF-α level could influence the sensitivity of GBM cells to radiotherapy. The result showed TNF-α inhibition could significantly suppress U251 and U87 cell proliferation when treated with different doses of irradiation (*P* < 0.01), while TNF-α stimulator promoted GBM cell proliferation under radiotherapy treatment (Fig. 6C).

### CARHSP1 mRNA levels correlate with survival in GBM patients with radiotherapy

**Fig. 7:**
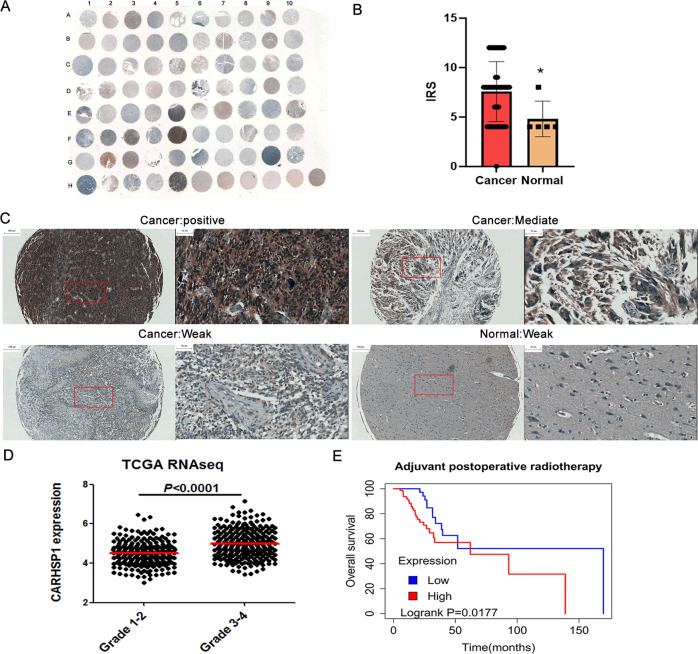
High CARHSP1 levels correlate with poorer survival in irradiation-treated GBM patients. **A** Overall staining of tissue microarray. **B** The IRS value of CARHSP1 protein in GBM tissues was significantly higher than that in adjacent normal brain tissues. **C** The positive, moderate, or weak immunostaining of CARHSP1 protein was shown in GBM tissues and the weak immunostaining of CARHSP1 protein in adjacent normal brain tissues. **D** High CARHSP1 expression was significantly associated with pathological grade in TCGA data. **E** Overall survival for irradiation-treated GBM patients (a total of 139 patients) showed high levels of CARHSP1 correlate with poorer survival.

The expression levels of CARHSP1 protein in GBM and adjacent normal brain tissues were examined by immunohistochemistry analysis. As a result, the positive, moderate or weak immunostaining of CARHSP1 protein was shown in GBM tissues and the weak immunostaining was shown in adjacent normal brain tissues (Fig. 7C). Statistically, the IRS value of CARHSP1 protein in GBM tissues was significantly higher than that in adjacent normal brain tissues (IRS, GBM, 7.6 ± 3.0 vs. benign, 4.8 ± 1.8, *P* < 0.05, Fig. 7B). In addition, high expression of CARHSP1 protein was associated with age (*P* = 0), pathological grade (*P* = 0) (Fig. 7D) and recurred (*P* = 0.0009) (Supplementary Table S3). The 139 irradiation-treated patients (Supplementary Table S4) were divided into CARHSP1 low and high expression groups according to the same threshold as the total survival analysis (4.6978) and then the survival analysis was conducted. Surprisingly, we found that irradiation-treated patients with high CARHSP1 mRNA levels had significantly poorer survival compared with patients with low CARHSP1 levels (Fig. 7E, *P* = 0.0177, log-rank test). These results suggest that CARHSP1 is a poor prognostic marker in GBM, more importantly, high CARHSP1 mRNA identifies patients less responsive to radiotherapy and could be prognostic of overall survival.

## Discussion

Glioblastoma (GBM) is one of the most aggressive brain tumors. Surgery followed by radiotherapy is the standard therapeutic regimen for GBM [9]. Upon treatment by radiation, the primary response of GBM cells is proliferation arrest and the arrested cells then undergo premature senescence within 4–8 days after irradiation [25]. Although the treatments could prolong survival of GBM patients, almost all patients eventually develop resistance. Therefore, there is a critical need to understand how radioresistance is acquired. The previous study [2, 26] has shown that mesenchymal signature, CD44 expression, NF-kB activation and induction of ER stress signaling correlated with poor radiation response and shorter survival in patients with GBM. There is still an urgent need to discover new therapeutic targets that can enhance the radiotherapy sensitivity of GBM.

The genome-wide CRISPR/Cas9 screening enables researchers to identify genes contributing to a specific phenotype. In this study, we adopted the transcriptional activation screening strategy using the SAM2 library to identify determinate genes that are essential for radioresistance in GBM as previously described [27, 28]. Here, we showed that the gain-of-function of CARHSP1, KIAA0895, FBMIL1, and STRA6 was highly associated with radioresistance in GBM. Further study showed that inhibition of the candidate genes sensitized GBM cells to radiotherapy and the expression of CARHSP1 mRNAs levels were significantly higher in irradiation-resistant GBM cells than that in wild-type GBM cells, which established a direct connection of CARHSP1 with radioresistance in GBM.

CARHSP1 is a ubiquitously expressed phosphoprotein that contains a cold-shock domain (CSD) with two RNA binding motifs [17, 29]. To date, there is limited information regarding CARHSP1, focusing primarily on the signaling pathways that phosphorylate and dephosphorylate. CARHSP1 [17, 30–32], as a regulator of TNF-α mRNA stability [21] and might be involved in oxidative stress via traffic between stress granules and processing bodies [18]. All living organisms must adapt to environmental changes, including cold shock, heat shock, and nutritional status. Previous data [33] had shown that a cold-shock proteins system could make *E. coli* resistant to the lethal effects of gamma rays. Park SH, et al’s study [34] showed the overexpression of cold-shock domain-containing protein PprM in *E. coli* significantly increased the tolerance to hydrogen peroxide (H_2_O_2_) and elevated the expression of oxygen-dependent genes, which might play important roles in oxidative stress tolerance. Interestingly, CARHSP1 is a CSD-containing protein with a high level of resemblance to cold-shock proteins, it’s overexpression might elevate the expression of oxygen-dependent genes and thus plays a pivotal role in modulating the radiation responsiveness of cells.

Several studies [10, 35–37] have produced evidence that radioresistance occurs through various cellular conditions including survival signaling activation, hypoxic microenvironments, autophagy, tumor stem cells, and inflammatory responses. Among these factors, inflammation plays a pivotal role in modulating the resistance of cancer cells to radiotherapy. And many inflammation mediators such as cytokines and chemokines play crucial roles in the growth and survival of cancer cells as well as the activation of oncogenic transcription factors including NF-κB [26]. Our study showed that the CARHSP1 mRNA level was upregulated in irradiation-resistant GBM cells. In addition, the CARHSP1 mRNA level was positively correlated with TNF-α. We deduced that a high level of CARHSP1 could lead to irradiation resistance by promoting the expression of TNF-α. In vitro validation experiments showed inhibition of TNF-α inhibition could significantly suppress GBM cells proliferation, while TNF-α stimulator promoted GBM cell proliferation under radiotherapy treatment. Mechanistically, a gain of CARHSP1 might facilitate radioresistance through promoting TNFα.

TNF-α is a pleiotropic cytokine and a central mediator of inflammation, and chronic inflammation mediated by TNF-α is also associated with tumor progression [38, 39]. It was proved that knockdown of CARHSP1 inhibits TNF-α protein production in LPS-stimulated cells and reduces the level of TNF-α mRNA in both resting and LPS-stimulated cells [21]. Moreover, the TNF-α/NF-kβ pathways were significantly activated following radiotherapy which indicated the activation of inflammatory genes upon radiation [40]. Thus, we concluded that the high expression of CARHSP1 upon radiation might mediate radioresistance by activating the TNF-α/NF-kβ pathway. More importantly, high CARHSP1 levels were correlated with poorer survival in irradiation-treated GBM patients according to the TCGA database. Targeting the CARHSP1/TNF-α inflammatory signaling induced by radiotherapy might directly affect radioresistance and present an attractive therapeutic target for GBM.

Collectively, these findings suggested that CARHSP1 contributes to in vitro resistance to radiotherapy in GBM cancer cells. We showed that patients with high levels of CARHSP1 had poorer survival when treated with radiotherapy. Targeting the CARHSP1/TNF-α inflammatory signaling activation induced by radiotherapy might directly affect radioresistance and presents an attractive therapeutic target for GBM, particularly for patients with high levels of CARHSP1.

## Supplementary information

Figure.S1

Figure.S2

Table S1

Table S2

Table S3

Table S4
