# Supplementary material for: Genome-wide CRISPR/Cas9 screening identifies CARHSP1 responsible for radiation resistance in glioblastoma
Source: Cell Death Dis. 2021 Jul 21;12(8):724. doi: 10.1038/s41419-021-04000-3 (PMC8295287; doi:10.1038/s41419-021-04000-3)
Supplement: Supplementary file 3 — Table S1 [file 41419_2021_4000_MOESM3_ESM.doc]

**Table. S1** Primers sequences for q-PCR.

| **Primer name** | **Product size** | **Sequences** |
| --- | --- | --- |
| β-actin | 185 bp | F：TGGCACCAGCACAATGAA |
|  |  | R：CTAAGTCATAGTCCGCCTAGAAGCA |
| CARHSP1 | 240bp | F：ACATTCTTCCACACGAGACGG |
|  |  | R：CCACCCTTGATCCACTCCCT |
| KIAA0895 | 170bp | F：TCCCAACCAGGGTGTTTTAGT |
|  |  | R：GGGACCCTCATGTTTTCGGTA |
| FBLIM1 | 223bp | F：AATCTGATCCCAGGTGAGGGAG |
|  |  | R：CCAGGGTGATAAAGACAGACGA |
| STRA6 | 141bp | F：TCCTATGGCAGCTGGTACATC |
|  |  | R：CAGCAGCACAAGGATTGACAG |
