# Supplementary material for: Genome-wide CRISPR/Cas9 screening identifies CARHSP1 responsible for radiation resistance in glioblastoma
Source: Cell Death Dis. 2021 Jul 21;12(8):724. doi: 10.1038/s41419-021-04000-3 (PMC8295287; doi:10.1038/s41419-021-04000-3)
Supplement: Supplementary file 5 — Table S3 [file 41419_2021_4000_MOESM5_ESM.doc]

**Table.S3** Associations of CARHSP1 mRNA expression with various clinicopathological characteristics in patients with GBM

| Clinical features | Case | x±s | P |
| --- | --- | --- | --- |
| Age |  |  |  |
| <50 | 341 | 4.6661±0.6594 | 0 |
| ≥50 | 158 | 4.9782±0.7462 |  |
| Sex |  |  |  |
| Female | 223 | 4.8146±0.7262 | 0.1565 |
| Male | 276 | 4.7247±0.6814 |  |
| Pathological grade |  |  |  |
| <3 | 241 | 4.5183±0.5963 | 0 |
| ≥3 | 257 | 5.0000±0.7147 |  |
| Overall survival |  |  |  |
| Alive | 374 | 4.6611±0.6682 | 0 |
| Dead | 125 | 5.0756±0.7139 |  |
| Disease free survival |  |  |  |
| Disease Free | 299 | 4.6516±0.6563 | 0.0009 |
| Recurred/Progressed | 163 | 4.8748±0.7289 |  |
